# Cardiac Phenotype and Gene Mutations in RASopathies

**DOI:** 10.3390/genes15081015

**Published:** 2024-08-02

**Authors:** Maria Felicia Faienza, Giovanni Meliota, Donatella Mentino, Romina Ficarella, Mattia Gentile, Ugo Vairo, Gabriele D’amato

**Affiliations:** 1 Pediatric Unit, Department of Precision and Regenerative Medicine and Ionian Area, University of Bari “Aldo Moro”, 70124 Bari, Italy; donatella.mentino@uniba.it; 2 Department of Pediatric Cardiology, Giovanni XXIII Pediatric Hospital, 70126 Bari, Italy; giovanni.meliota@gmail.com (G.M.); ugovairo1@gmail.com (U.V.); 3 U.O.C. Laboratorio di Genetica Medica, PO Di Venere-ASL Bari, 70012 Bari, Italy; romina.ficarella@als.bari.it (R.F.); mattia.gentile@asl.bari.it (M.G.); 4 Neonatal Intensive Care Unit, Di Venere Hospital, 70012 Bari, Italy; gabriele.damato@asl.bari.it

**Keywords:** RASopathies, Noonan syndrome, congenital heart disease (CHD), hypertrophic cardiomyopathy (HCM), pulmonary valvular stenosis (PVS)

## Abstract

Cardiac involvement is a major feature of RASopathies, a group of phenotypically overlapping syndromes caused by germline mutations in genes encoding components of the RAS/MAPK (mitogen-activated protein kinase) signaling pathway. In particular, Noonan syndrome (NS) is associated with a wide spectrum of cardiac pathologies ranging from congenital heart disease (CHD), present in approximately 80% of patients, to hypertrophic cardiomyopathy (HCM), observed in approximately 20% of patients. Genotype–cardiac phenotype correlations are frequently described, and they are useful indicators in predicting the prognosis concerning cardiac disease over the lifetime. The aim of this review is to clarify the molecular mechanisms underlying the development of cardiac diseases associated particularly with NS, and to discuss the main morphological and clinical characteristics of the two most frequent cardiac disorders, namely pulmonary valve stenosis (PVS) and HCM. We will also report the genotype–phenotype correlation and its implications for prognosis and treatment. Knowing the molecular mechanisms responsible for the genotype–phenotype correlation is key to developing possible targeted therapies. We will briefly address the first experiences of targeted HCM treatment using RAS/MAPK pathway inhibitors.

## 1. Introduction

RASopathies are a group of syndromes with overlapping features caused by germline mutations in genes encoding for components of the RAS/MAPK (mitogen-activated protein kinase) signaling pathway which regulates cell growth, differentiation, proliferation, migration, and apoptosis. Dysregulation of RAS/MAPK signaling may be due to the upregulation of RAS GTPases or RAS effectors belonging to the MAPK cascade, increased activity of proteins that positively regulate RAS function or signaling transmission to downstream transducers, or alterations in the switch-off of signaling controlled by feedback mechanisms [1]. RASopathies include Noonan syndrome (NS), cardio-facio-cutaneous syndrome (CFCS), Costello syndrome (CS), Noonan syndrome with multiple freckles (NSML) or LEOPARD syndrome (LS), the Noonan-like syndrome with loose anagen hair (Mazzanti syndrome), Legius syndrome, Neurofibromatosis–Noonan syndrome (NF/NS), CBL mutation syndrome, and a rising number of clinically related disorders [2]. Each of these conditions has distinctive features, although they share overlapping characteristics. Among RASopathies, NS has an incidence varying from 1:1000 to 1:2500 live births [2]. It was first reported by Jacqueline Noonan as a syndrome characterized by pulmonary valvular stenosis (PVS) and multiple extracardiac features [3]. These include distinctive facial dysmorphic features (low-set ears, hypertelorism, ptosis), short stature, chest deformities, cryptorchidism, delayed puberty, neurodevelopmental disabilities, bleeding disorders, and risk for hematologic and solid cancers [4,5]. NS is a disorder predominantly transmitted in an autosomal dominant manner with a high proportion of de novo mutations, although an autosomal recessive form has been recently identified [6]. Establishing the diagnosis of NS can be difficult due to the large variability in the phenotype, which becomes less pronounced with increasing age. A scoring system has been proposed to facilitate the clinical diagnosis of NS [7].

With the genetic characterization of NS, significant genotype–phenotype correlation has been found for the numerous genes that cause this syndrome [8]. The first gene identified for NS and causative of the condition in approximately 50% of affected individuals was protein tyrosine phosphatase, the non-receptor type 11 (*PTPN11*) gene that encodes Src homology 2-containing protein tyrosine phosphatase 2 (SHP2), a ubiquitous cytoplasmic protein that modulates various intracellular signaling and several growing processes [9]. The enormous effort in discovering the genes associated with RASopathies in the last 20 years led to the identification of more than 20 genes implicated in NS, such as *SOS1*, *RAF1*, *RIT1*, *KRAS*, *NRAS*, *BRAF*, *LZTR1*, *SOS2*, and others [1,10]. The causal mutations remain unrevealed in 10–20% of patients. 

Cardiac involvement is one of the principal characteristics of NS; indeed, affected subjects have a broad spectrum of cardiac diseases, belonging to two main types: congenital heart disease (CHD), found in ~80% of patients, and hypertrophic cardiomyopathy (HCM), observed in ~20% of patients [11,12,13]. Although PVS and HCM represent the defects most frequently observed, a variety of cardiac malformations such as atrioventricular canal defect (AVCD), mitral valve (MV) anomalies, atrial septal defect (ASD), aortic coarctation, and hypoplastic left heart syndrome (HLHS) have been progressively included in the phenotypic spectrum of heart diseases associated with RASopathies [14] (Table 1). 

After the identification of genes associated with these syndromes, it was possible to identify a genotype–phenotype correlation with respect to the majority of CHD. Atypical CHDs in RASopathies have a less defined genetic profile. 

This review aims to elucidate the molecular mechanisms underlying heart disease development in RASopathies and to discuss the main morphological and clinical features of the two most frequent cardiac disorders, namely PVS and HCM. We will report genotype–phenotype correlation and its implication in prognosis and treatment.

## 2. Molecular Involvement of RAS/MAPK Signaling Pathway in the Development of Heart Diseases

Dysregulation of the RAS/MAPK pathway due to pathogenetic mutations in genes involved in RASopathies determines cardiovascular defects associated with these disorders (Table 2) [14,15]. 

*PTPN11* is the gene most frequently involved in RASopathies, accounting for 50% of all NS cases, and 85% of patients with LS. It encodes for the protein tyrosine phosphatase SHP2, which has a critical role in normal cardiac development and function. SHP2 is required for cardiac progenitor cell differentiation and proliferation, and it acts through the activation of distinct signaling pathways: mitogen-activated protein kinase/extracellular signal-regulated kinase (MAPK/ERK), Fibroblast Growth Factor/Bone Morphogenetic Protein (FGF/BMP,) calcineurin–Nuclear Factor of Activated T-cell (NFAT)-dependent signaling, vascular endothelial growth factor (VEGF), Notch transmembrane receptors, Wingless-Type Mmtv Integration Site Family (Wnt)/β-catenin, transforming growth factor-β/Bone Morphogenetic Protein (TGF-β/BMP), ERBB family of receptor tyrosine kinases, and the Mammalian Target Of Rapamycin/Ser/Thr kinase AKT (mTOR/AKT) signaling cascade. Its role has been demonstrated in different SHP2-mutated animal models, which show cardiac phenotypes such as Xenopus [16], Zebrafish [17], and several mouse models of NS (Ptpn11^D61G/+^ mice), showing enlarged cushions, ASD, VSD, double-outlet right ventricle (DORV), myocardial thinning with no evidence of HCM [18], and LS mutants (Q510E-Shp2 mice), showing HCM and enlarged atria [19]. Studies in vivo and in vitro demonstrated different biochemical mechanisms between the PTPN11-NS and LS mutations. SHP2 mutants, causative of NS, display an increased basal activity without affecting PTP enzymatic activity, and are classified as hypermorph alleles (gain-of-function alleles) that can augment ERK/MAPK pathway activation. In contrast, LS mutations seem inactive, with reduced and/or absent PTP catalytic activity, resulting in a *loss-of-function (LOF) allele* of the phosphatase and enhanced AKT/mTOR activity. Therefore, LS mutations primarily affect the AKT/mTOR signaling pathway, and not the ERK/MAPK pathway as in NS. According to these observations, the inhibition of the mTOR signaling by rapamycin, a drug that specifically targets and suppresses TOR activity, is able to reverse and normalize the LS cardiac phenotype [16]. Everolimus, a rapamycin analog, has recently been used for compassionate use in vivo with promising results [20].

Cardiac hypertrophy has also been reported in H-Ras-mutated in vitro and in vivo models. In detail, the oncogenic mutant G12V (Val12-Ras), the most common mutation in Costello syndrome, determines the transcriptional activation of the pro-hypertrophic transcription factor NFAT in a neonatal rat cardiomyocyte in vitro model (NRCM), causing hypertrophy with sarcomeric interruption and inhibition of cardiomyocyte beating, as well as an in vivo mouse model [21]. The main molecular signaling event involved in the pathogenesis of cardiac hypertrophy is the activation of the Ras/Raf/MEK/MAPK pathway and the downstream molecular cascades, the ERK and the Calcineurin/NFAT pathways, with consequent modulation of specific transcription factors such as MEF2, JUN, and GATA4, which produce the expression of genes involved in the pathogenesis of hypertrophy. Another in vivo mouse model underlined the potential role of K-Ras, which is mutated in less than 5% of NS patients, in cardiac hypertrophy. A K-Ras^V14I^ mouse model carrying V14I, a germ-line mutation recurrent in K-Ras-mutated-NS patients, developed heart anomalies, such as cardiac hyperplasia due to a greater number of cardiomyocytes, probably caused by an expansion of the cardiac progenitor cells [22]. 

Germline mutations in *RAF1* are found in 3–5% of NS subjects. Raf1^L613V^ mice show normal valvuloseptal growth, but exhibit eccentric cardiac hypertrophy, probably due to enhanced MEK-ERK signaling, confirming the relevant role of the RAS-ERK pathway in the pathogenesis of HCM [23]. To investigate the pathogenetic mechanism of *RIT1* mutations in NS, knock-in mice carrying the A57G mutation (Rit1^A57G^) were analyzed, which exhibited cardiac hypertrophy, cardiac fibrosis, and other NS features [24]. Different findings were observed in an *Nras^G12D^* in vivo mouse model: the mutant embryos exhibited cardiac malformations including VSD, DORV, and PVS, the common CHDs characterizing NS, resulting from the hyperactivation of the RAS/RAF/MEK/ERK pathway and downstream signaling molecules, including ERK and AKT [25]. Focusing on cardiac phenotype, functional analysis has also been reported for other genes, such as *SOS1* and *BRAF*, in order to provide evidence of their role in determining heart defects. Mice with the NS-associated Sos1^E846K^ gain-of-*function* mutation showed NS-associated phenotypes, including growth delay, hematologic abnormalities, and cardiac defects such as left ventricular hypertrophy with incomplete aortic stenosis, ventricular and epicardial fibrosis, and adipocyte infiltration. The Ras/MAPK pathway and downstream Rac1 and Stat3 molecules, already associated with cardiac disease and heart failure, are presumably involved [26]. Regarding the *Braf* gene, two mouse models clarify the role of this gene in the cardiac phenotype of NS and CFC syndrome. Braf^L597V^ mice expressing an L597V mutation, causative of NS, developed typical features, including short stature and cardiac enlargement as well as an increased cardiomyocyte cross-sectional area, indicative of cardiac hypertrophy, while Braf^Q241R^, expressing the Braf Q241R mutation, which is the most frequent gain-of-function mutation in CFC syndrome, showed cardiomegaly, expanded cardiac valves, ventricular noncompaction, and VSDs, through activation of the RAS–MAPK pathway, as established by using MEK inhibitors [27]. The first evidence of the efficacy of MEK inhibition in humans with trametinib opens new perspectives in the treatment of patients with RASopathies, as later discussed in paragraph 5. Among the emerging conditions in RASopathy spectrum disorders, there is the Mazzanti syndrome, an NS-like disorder with loose anagen hair caused by mutations in the *SHOC2* variant associated with prenatal-onset HCM. By using an in vitro cellular system, it has been demonstrated that both SHOC2 p.Gln269_His270delinsHisTyr and the recurrent p.Ser2Gly pathogenic mutations promote augmented binding of the mutant protein to MRAS and PPP1CB and increased MAPK signaling cascade [28].

## 3. Heart Diseases Associated with RASopathies

**Figure 1:**
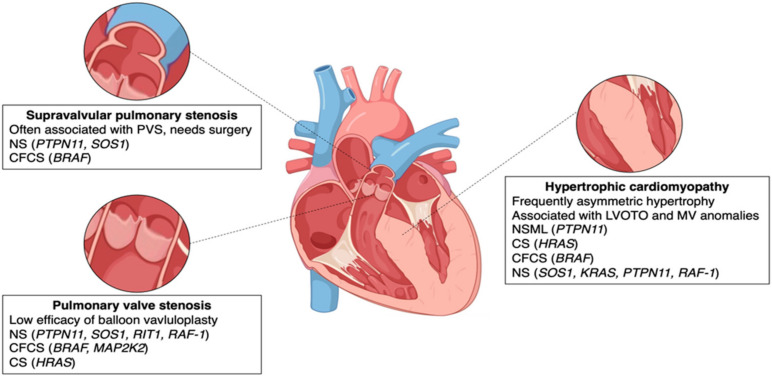
The most frequent cardiac diseases in NS and RASopathies are valvular and supravalvular stenosis and hypertrophic cardiomyopathy. The syndromes and the related involved genes are listed in order of frequency. NS: Noonan syndrome; CFCS: cardio-facio-cutaneous syndrome; CS: Costello syndrome; LOVOTO: left ventricular outflow obstruction; MV: mitral valve; NSML: Noonan syndrome with multiple lentigines; PVS: pulmonary valve stenosis.

PVS represents the most frequent NS-associated CHD with an estimated prevalence of ~40%, but other types have been described, particularly ASD (8%) or ventricular septal defects (VSDs), as well as AVCDs (15%) [12,13]. Less frequently, patients show left-sided forms of CHD including MV stenosis (6%), aortic valve stenosis, and aortic coarctation (9%) [29] (Table 1). The most severe form of left heart obstruction, hypoplastic left heart syndrome, has only been reported anecdotally [30]. Infrequently, tetralogy of Fallot or patent ductus arteriosus is detected in NS. Often, patients will display complex cardiac phenotypes with multiple defects such as PVS and AS or CHD and HCM [14]. The most common conditions associated with NS and RASopathies are PVS and HCM, which have distinctive features compared to non-syndromic patients (Figure 1).

In patients with RASopathies, genotype–phenotype correlations are frequently described, and they are useful indicators in predicting the prognosis concerning cardiac disease over the lifetime (Figure 1). Patients with *PTPN11*-associated NS are distinguishable from patients with *RAF1* variants, and they have a dissimilar risk for cardiovascular disease, with *PTPN11* presenting a higher risk for PVS and less risk for HCM. The opposite is true for *RAF1*. Variants in the same gene are responsible for different RASopathies and cardiac phenotypes. While a mutation on codon 308 of the *PTPN11* gene is associated with severe PS in NS, exon 7, 12, and 13 variants of the *PTPN11* gene are associated with HCM in NSML [31]. The management of heart disease in RASopathies depends on the specific cardiac condition. When it comes to cardiac outcomes, cardiac abnormalities can differ greatly in terms of their phenotype and severity. As a result, their clinical involvement is highly variable. CHD patients with RASopathies generally have good long-term outcomes and a low overall mortality rate of less than 2.5% in the general population, which is less than 3% in the subset with cardiac disease. Death occurs typically in the first two years of life or adulthood [12]. On the other hand, when dealing with HCM, morbidity and mortality are significantly higher in patients with RASopathies [32].

### 3.1. Pulmonary Stenosis

**Figure 2:**
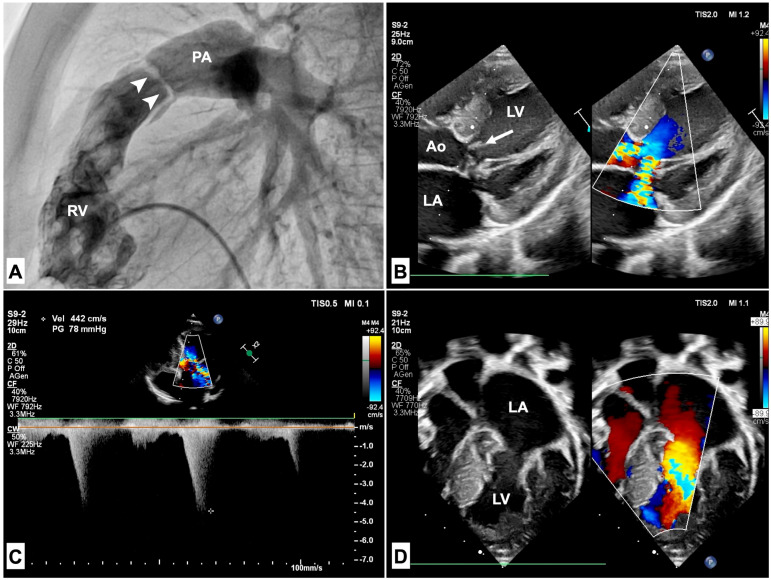
(**A**): RV angiography shows a pulmonary valve (arrows) with thickened leaflets, doming systolic motion, and tight orifice, causing severe pulmonary valve stenosis. The PA trunk is not dilated, as the valve is characterized mostly by dysplasia rather than a fusion of commissures. (**B**,**C**): Echocardiographic findings of HCM in an NSML patient. The ventricular septum is sigmoid with a septal bulge. During systole, anterior systolic motion of the anterior mitral leaflet (arrow) causes severe LVOTO and mitral regurgitation, with a peak gradient of nearly 80 mmHg (**C**). (**D**): In an NS patient with HCM, severe LV hypertrophy is associated with a biconvex shape of the ventricular septum. Anomalous insertion of mitral chordae is also displayed. Ao: aorta; LA: left atrium; LV: left ventricle; PA: pulmonary artery; RV: right ventricle.

Pulmonary stenosis (PS) is the most frequent CHD, with a prevalence of about 70% of patients with NS [33], mainly due to PTPN11 variants [34]. The obstruction is usually at a valvular level with peculiar anatomic features, as the pulmonary valve (PV) typically presents a dysplastic phenotype with myxomatous thickening. The valvular leaflets appear deformed, glistening, and edematous, with poor systolic motion. A typical feature of PVS in non-syndromic patients is the fusion of the commissures of the pulmonary valve. In patients with RASopathies, this aspect is seldom absent and the valve is stenotic mainly due to its dysplastic features (Figure 2A) [35]. It has therapeutic implications, as balloon valvuloplasty acts more effectively on a valve with fusion of the commissures rather than on thickened cusps. The degree of PS severity is variable, with approximately one-third of PVS patients having a severe obstruction and 60% having only mild stenosis [36]. Mild PVS is usually non-progressive and does not require treatment. Moderate-to-severe stenosis, on the other hand, is associated with a higher rate of intervention due to greater dysplasia of the valve leaflets (Figure 2A). Frequently, a supravalvular stenosis is described, with a concomitant fusion of valvular cusps with the vessel wall [37]. An association between phenotype and genotype has been described. Usually, a mutation on codon 308 of the *PTPN11* gene is associated with severe PS, both at valvular and supravalvular levels [38]. Patients with *SOS1* and *SOS2* variants usually have a mild PVS associated with ASD [39,40]. The distinct anatomic PS features have an impact on the treatment efficacy. Compared with non-syndromic patients with pulmonary stenosis, the early outcome associated with percutaneous balloon valvuloplasty is poorer and NS patients frequently need reintervention or surgery [41,42,43]. In a 5-year follow-up study of patients with congenital PS who underwent balloon pulmonary valvuloplasty, 80% of NS patients had a suboptimal result, defined as an immediate post-intervention PV gradient > 20 mmHg, instead of 15% for non-syndromic patients. However, the pulmonary valve gradient of NS patients continually decreased over time [44]. Data from the VACA registry, a multi-institutional registry of 533 patients who underwent balloon pulmonary valvuloplasty, showed that dysplastic pulmonary valves, which are typically described in NS, were an independent risk factor for a poor response to balloon valvuloplasty [42]. In another retrospective study, 50% of NS patients with PS required reintervention 28 ± 54 months after the first balloon valvuloplasty [45]. In another retrospective study, 50% of NS patients with PS required reintervention 28 ± 54 months after the first balloon valvuloplasty. These patients had a lower PV gradient reduction after balloon dilation and showed a steady gradient during follow-up. Supravalvular stenosis is a risk factor for reintervention in the case of concomitant PVS requiring balloon dilation, even in the case of a tiny membrane with a small contribution to the right ventricular-to-pulmonary artery gradient [46]. 

When more than moderate supravalvular obstruction is present, surgical repair is needed [43]. There is currently a gap in knowledge for adults with RASopathies and PVS. Pierpont and Digilio [47] report that in their cohort, nearly 50% had heart surgery, and about 3.5% had a clinically significant arrhythmia. Long-term complications of pulmonary regurgitation may be expected in patients with PVS following surgical or catheter treatments. Regardless of the presence of poor data in the literature, the management of these patients should be comparable to non-syndromic ones. Transcatheter or surgical pulmonary valve replacement should be indicated later in life, in case of symptoms or progressive right ventricular dysfunction [48].

### 3.2. Hypertrophic Cardiomyopathy

RASopathies are a frequent cause of HCM in infancy and childhood. They are reported as an underlying etiology in up to 42% of infants with HCM in recent pediatric longitudinal cohorts [48,49]. The incidence of HCM varies across RASopathies. NSML has the highest prevalence among RASopathies, with an HCM diagnosis in approximately 80% of patients [33]. Prevalence is lower in other conditions, being 65% in Costello syndrome, 40% in CFC, and 20–25% in NS [50]. NS patients also seem to present a higher risk of dilated cardiomyopathy in adult life [51]. While *PTPN11* mutations are typically associated with PS in NS, in NSML, *PTPN11* variants are responsible for HCM [52]. Exon 13 variants of the *PTPN11* gene are associated with severe obstructive HCM [33]. Compared with non-syndromic conditions, at presentation in infancy, HCM in RASopathies has a more severe left ventricular hypertrophy and a higher prevalence of left ventricular outflow obstruction (LVOTO) [53,54], associated MV anomalies, and biventricular hypertrophy (Figure 2B–D). The displacement of papillary muscles, the anomalous insertion of mitral chordae (Figure 2D), and the systolic anterior motion of the MV are usually responsible for LVOTO (Figure 2B).

The MV anomalies may be associated with significant mitral regurgitation, causing anticipation of heart failure symptoms [55]. Moreover, the presence of MV anomalies is a marker of poorer prognosis and has been associated with a higher risk of reintervention and death [12]. The ventricular septum morphology may differ among RASopathies [56]. In NSML, it appears sigmoid with a septal bulge (Figure 2B), while in NS, it is more frequently observed as a biconvex shape (Figure 2D) [57]. Myocardial ischemia is frequently observed. It is a significant clinical problem in adolescents and young adults with RASopathies [33]. Coronary artery anomalies, which have been reported in up to 30% of patients [14], may contribute to myocardial ischemia. The association of HCM and PVS is not rare, and it is characterized by biventricular hypertrophy. This association is described in up to 65% of NS with HCM, and it is related to poorer prognosis [50]. Biventricular hypertrophy is reflected by an extreme right axis deviation (a “superior” QRS axis) on the electrocardiogram and represents a specific disease marker [58]. Other ECG abnormalities reported are pseudo-infarction q waves and prolonged QT interval. In patients with RASopathies, atrial arrhythmias are quite common; however, in patients with Costello syndrome, they have a higher prevalence and may be detected in up to 56% of cases [59]. Therefore, focal atrial tachycardia may serve as a diagnostic hint for underlying Costello syndrome. Moreover, *RAF1* mutations are specifically associated with multifocal atrial tachycardia [60]. A child with HCM should always receive a thorough first examination that includes methodical research for clinical and instrumental clues. Some “red flags” such as facial dysmorphism, lentigines, sensorineural deafness, PVS, biventricular hypertrophy, and extreme right axis deviation at ECG may indicate the diagnosis of RASopathy [61]. 

HCM is a major determinant of the clinical prognosis, as it is related to earlier mortality (22% at 1 year) and earlier onset of heart failure symptoms [62]. 

In contrast to non-syndromic primary HCM, HCM in RASopathies confers a high risk of mortality in infancy, which could be attributed to earlier age at presentation and occurrence of heart failure [63]. 

In an international multicenter retrospective cohort study of pediatric patients with primary HCM and RASopathy-related HCM (the PRIMaCY study), heart failure was the leading cause of mortality during infancy in patients with RASopathy, and sudden cardiac death emerged as the leading cause of mortality in adolescent and teenage RASopathy patients. RASopathy patients had a 50% higher risk (11% vs. 5.4%) of nonarrhythmic death or transplant at 10 years after first evaluation, with most deaths in the RASopathy cohort occurring in infancy. The major contributor to mortality in this group was heart failure, accounting for 28% of all deaths during follow-up. The overall burden of sudden cardiac death was not different in the two groups, with a 10-year cumulative incidence of 4.7% in RASopathy HCM and 4.2% in primary HCM [64].

This emphasizes the unacknowledged burden of sudden cardiac death and the necessity of routine surveillance for sudden mortality risk in older patients with HCM and RASopathies. To date, there is a gap of knowledge in the literature about risk stratification for sudden cardiac death in patients with HCM and RASopathies. In an international multicenter observational cohort study of 572 patients with HCM [65], a sudden cardiac death score was validated, but RASopathies were excluded from the analysis. In a recent validation study for study for the HCM Risk-Kids model in patients with RASopathy-associated HCM, the model showed a very low specificity and positive predictive value, as the majority of patients who had a sudden cardiac death equivalent event had a low estimated 5-year SCD risk [66].

As the PRIMaCY study showed, a 5-fold lower implantable cardioverter–defibrillator (ICD) use in patients with RASopathies compared to P-HCM patients despite similar age and incidence of SCD events in adolescents and teenagers. This emphasizes the necessity of developing novel risk prediction models for sudden cardiac death specific to individuals with RASopathies and HCM in order to support primary prevention ICD decision-making. General management of HCM is based on current clinical practice guidelines. Non-vasodilating β-blockers, alone or in association with disopyramide, are generally used to relieve symptoms and the degree of LVOTO. Disopyramide has also proven to be effective in NS, but its effect is generally temporary [67]. Surgical septal myectomy should only be reserved for patients with severe symptomatic obstruction, refractory to optimal medical therapy [68]. Orthotopic heart transplantation (HT) is a rare procedure that may be considered for people with advanced heart failure, refractory ventricular arrhythmias, or severe diastolic dysfunction [69]. Both cardiac and noncardiac risk should be considered when evaluating HT indications. Patients with NS-linked *PTPN11* and *RIT1* mutations are known to be at risk for coagulopathy. There is an increased risk of cancer with other mutations. In such cases, the comorbidities can have an impact on early and late HT outcomes and therefore must be taken into consideration.

## 4. Discussion

Cardiac involvement in NS and RASopathies is frequent and influences the quality of life and prognosis of these individuals. Although the outcome of CHD is substantially comparable to non-syndromic patients, the peculiar pathologic characteristics make standard treatments less effective. That is the case of percutaneous valvuloplasty in PVS. Moreover, associated extracardiac comorbidities may influence peri-procedural outcomes. Lymphatic abnormalities are associated with chylothorax in up to 10% of RASopathy patients who underwent surgical repair [43]. Bleeding abnormalities, including coagulation factor deficiency and platelet dysfunction, have a prevalence of 50–89% [70]. Investigating the bleeding history and the coagulation system in these individuals is therefore crucial, as they can lead to possible surgical hemorrhagic complications [71]. HCM is the cardiac disease with the highest impact on the prognosis of RASopathy patients. The co-presence of HCM and CHD makes survival even worse. Selected cases may require heart transplantation. Continuous lifelong cardiac surveillance is advisable to exclude long-term sequelae after major cardiac procedures. It can also provide data on adult prognosis and risk stratification of sudden cardiac death in HCM. Such information is still lacking in the current literature. Identifying genotypes is crucial for clinical prognosis concerning cardiac disease, neurodevelopment, and other organ system involvement. This information should play a significant role in counseling the family after a diagnosis of RASopathy. Future research will rely on the capacity to predict hospital and surgical outcomes based on the genotype. Trametinib and sirolimus are two experimental RAS/MAPK pathway inhibitors investigated for their potential use in treating HCM and other comorbidities, including lymphatic disease and cancer. 

It will be essential to study the impact of different gain-of-function variations in the RAS/MAPK pathway to determine the efficacy of these therapies in children with NS and other RASopathies. Until genetic testing becomes universal for CHD patients, attention to the broad spectrum of NS features remains crucial. Clinicians should pay attention to the clinical and instrumental red flags of RASopathies and orient their suspicion to specific genetic testing.

## 5. Future Directions

The most important point to consider is that diagnoses of RASopathies are generally made after birth, but in some cases, the suspicion may be formulated prenatally, based on the detection of abnormal ultrasound findings such as severely increased nuchal translucency, cystic hygroma, and/or cardiac abnormalities. A timely diagnosis allows for an accurate prognosis and, above all, optimal neonatal cardiac management. Based on evidence from in vivo translational studies of the involvement of RAF/MEK/ERK and subsequent downstream signaling pathways (such as PI3K/Akt/mTOR and Ca^++^/Calcineurin/NFAT) on cardiac defects, they have become potential therapeutic targets to treat heart failure in RASopathy patients, both with pharmacological and non-pharmacological therapies (mRNA silencing), focusing on the genotype, which is also useful for predicting the clinical outcome. There is an increased risk of cancer with other mutations. Knowing the molecular mechanisms responsible for the genotype–phenotype correlation is key to developing possible targeted therapies. In patients with RASopathies and HCM, an increased activation through the RAS-MAPK cascade should be responsible for cardiomyocyte hypertrophy and myocardial fiber disarray [52]. MEK1/2 inhibition is approved to treat specific cancers with activation of the RAS-MAPK pathway [72]. In a Raf-mutated murine model of NS, Mek1 inhibition during 4–10 weeks of life seemed to ameliorate the cardiac phenotype [23]. In a recent clinical report, two neonates with severe HCM and PVS had an *RIT1* mutation. They received therapy with Trametinib, a highly selective reversible allosteric inhibitor of MEK1/2 activity [73]. After 4 months, the treatment was associated with reversal of progressive myocardial hypertrophy and valvular obstruction. Clinical evidence of myocardial hypertrophy reversal was reported in another newborn with NS and severe HCM with refractory heart failure treated with Trametinib [74]. In NSML, specific *PTPN11* missense mutations seem to increase activity through the mTOR–PI3K–AKT signaling pathway. In a *PTPN11*-mutant murine NSML model, treatment with rapamycin, an mTOR pathway inhibitor, ameliorated myocardial hypertrophy [75]. Recently, a 24-week-old infant with NSML and severe progressive HCM was treated with everolimus to prevent acute decompensation of heart failure before heart transplantation, but no reversal of cardiac hypertrophy was observed [20]. 

Larger studies and clinical trials are not yet available in the literature. Although they are necessary before the extensive use of these medications in clinical practice, there are some issues to overcome when considering the implementation of clinical trials, such as determining the best therapeutic targets (HCM, growth retardation, neurocognitive impairment), choosing the inclusion criteria concerning the RASopathy genotype, and considering the potential side effects of long-lasting therapy [76].

## Figures and Tables

**Table 1:** Congenital Heart Defects associated with RASopathies.

Type of Defects	Congenital Heart Defects
Right heart obstructive lesions	Pulmonary valve stenosis
	Supravalvular pulmonary stenosis
Shunt lesions	Atrial septal defect
	Ventricular septal defect
	Complete AV canal defect
	Partial AV canal defect
	Patent ductus arteriosus
Left heart obstructive lesions	Aortic valve anomalies
	Aortic coarctation
	Hypoplastic left heart syndrome
Complex cyanotic lesions	Tetralogy of Fallot
Other	Mitral valve anomalies
	Coronary artery anomalies

**Table 2:** Genes associated with RASopathies, main cardiac phenotypes, and molecular pathways involved in heart development.

Gene	Cardiac Phenotype	Pathway
*PTPN11*	PVS, HCM	Ras/Raf/MEK/MAPK, AKT/mTOR, FGF/BMP, TGF-β/BMP, WnT/β-catenin
*HRAS*	HCM	Ras/Raf/MEK/MAPK, Calcineurin/NFAT
*KRAS*	HRAS	Ras/Raf/MEK/MAPK,
*NRAS*	PVS, septal defects	Ras/Raf/MEK/MAPK,
*RAF1*	HCM	Ras/Raf/MEK/MAPK,
*RIT1*	HCM	Ras/Raf/MEK/MAPK,
*SOS1*	PVS, HCM, septal defects	Ras/Raf/MEK/MAPK,
*BRAF*	PVS, HCM, septal defects	Ras/Raf/MEK/MAPK,
*SHOC2*	HCM	Ras/Raf/MEK/MAPK,

Abbreviations: PVS, pulmonary valve stenosis; HCM, hypertrophic cardiomyopathy; MAPK/ERK, mitogen-activated protein kinase/extracellular signal-regulated kinase; AKT/mTOR, Mammalian Target of Rapamycin/Ser/Thr kinase AKT; FGF/BMP, Fibroblast Growth Factor/Bone Morphogenetic Protein; TGF-β/BMP, transforming growth factor-β/Bone Morphogenetic Protein; WnT/β-catenin, Wingless-type Mmtv Integration Site Family (Wnt)/β-catenin.; HRAS: Harvey Rat sarcoma virus; KRAS: Kirsten rat sarcoma virus.
